# A Member of the Ferlin Calcium Sensor Family Is Essential for Toxoplasma gondii Rhoptry Secretion

**DOI:** 10.1128/mBio.01510-18

**Published:** 2018-10-02

**Authors:** Bradley I. Coleman, Sudeshna Saha, Seiko Sato, Klemens Engelberg, David J. P. Ferguson, Isabelle Coppens, Melissa B. Lodoen, Marc-Jan Gubbels

**Affiliations:** a Department of Biology, Boston College, Chestnut Hill, Massachusetts, USA; b Department of Molecular Biology & Biochemistry and the Institute for Immunology, University of California, Irvine, California, USA; c Nuffield Department of Clinical Laboratory Science, University of Oxford John Radcliffe Hospital, Oxford, United Kingdom; d Department of Molecular Microbiology and Immunology, Johns Hopkins University Bloomberg School of Public Health, Baltimore, Maryland, USA; University of Arizona

**Keywords:** *Toxoplasma gondii*, calcium, ferlin, micronemes, protein secretion, rhoptries

## Abstract

Invasion of host cells by apicomplexan parasites such as Toxoplasma gondii is critical for their infectivity and pathogenesis. In Toxoplasma, secretion of essential egress, motility, and invasion-related proteins from microneme organelles is regulated by oscillations of intracellular Ca^2+^. Later stages of invasion are considered Ca^2+^ independent, including the secretion of proteins required for host cell entry and remodeling from the parasite’s rhoptries. We identified a family of three Toxoplasma proteins with homology to the ferlin family of double C2 domain-containing Ca^2+^ sensors. In humans and model organisms, such Ca^2+^ sensors orchestrate Ca^2+^-dependent exocytic membrane fusion with the plasma membrane. Here we focus on one ferlin that is conserved across the Apicomplexa, T. gondii FER2 (TgFER2). Unexpectedly, conditionally TgFER2-depleted parasites secreted their micronemes normally and were completely motile. However, these parasites were unable to invade host cells and were therefore not viable. Knockdown of TgFER2 prevented rhoptry secretion, and these parasites failed to form the moving junction at the parasite-host interface necessary for host cell invasion. Collectively, these data demonstrate the requirement of TgFER2 for rhoptry secretion in Toxoplasma tachyzoites and suggest a possible Ca^2+^ dependence of rhoptry secretion. These findings provide the first mechanistic insights into this critical yet poorly understood aspect of apicomplexan host cell invasion.

## Abstract (precis)

Apicomplexan protozoan parasites, such as those causing malaria and toxoplasmosis, must invade the cells of their hosts in order to establish a pathogenic infection. Timely release of proteins from a series of apical organelles is required for invasion. Neither the vesicular fusion events that underlie secretion nor the observed reliance of the various processes on changes in intracellular calcium concentrations is completely understood. We identified a group of three proteins with strong homology to the calcium-sensing ferlin family, which are known to be involved in protein secretion in other organisms. Surprisingly, decreasing the amounts of one of these proteins (TgFER2) did not have any effect on the typically calcium-dependent steps in invasion. Instead, TgFER2 was essential for the release of proteins from organelles called rhoptries. These data provide a tantalizing first look at the mechanisms controlling the very poorly understood process of rhoptry secretion, which is essential for the parasite’s infection cycle.

## INTRODUCTION

The apicomplexan parasite Toxoplasma gondii infects one in every three humans. Clinical symptoms of toxoplasmosis derive from the tissue destruction and inflammation caused by repeated rounds of host cell invasion, intracellular replication, and lytic egress of the tachyzoite life stage. Egress is mediated by intracellular Ca^2+^ ([Ca^2+^]_i_) fluctuations that trigger release of proteins from the microneme organelles (1). Following egress, parasites move via gliding motility to a new host cell, triggered by additional parasite [Ca^2+^]_i_ oscillations that facilitate further release of micronemes (2). Subsequent host cell invasion also relies on micronemal proteins (3). The micronemes are localized at the apical end of the parasite and are released from the apical tip (4). Following initial recognition of a host cell, the parasite engages in a tighter interaction with the target cell, mediated by proteins secreted from the rhoptries. The club-shaped rhoptries are anchored at the parasite’s apical end, from where they secrete their contents (5, 6). Proteins in the apical rhoptry neck (RONs) are secreted into the host cell before the rhoptry bulb proteins (ROPs) are released (7). RONs function in tightening the parasite-host attachment by forming a moving junction (MJ), whereas ROPs modulate a variety of host cell pathways to accommodate intracellular replication (6). Rhoptry secretion must be preceded by microneme secretion and requires recognition of a host cell. Although the molecular details of the underlying signal transduction pathways and the mechanism of rhoptry exocytosis remain obscure (8), rhoptry release is generally assumed to be Ca^2+^ independent. The last step in establishing infection of a new cell is secretion of host cell-remodeling proteins from the parasite’s dense granules, which again is believed to be Ca^2+^ independent.

The Ca^2+^ signal during egress and invasion is transduced by several molecular mechanisms, including calmodulin (2), calcineurin (9), Ca^2+^-dependent protein kinases (CDPK1 [10] and CDPK3 [11–13]), and at the site of microneme exocytic membrane fusion by the T. gondii DOC2.1 protein (14), referred to as TgDOC2 here. Unlike other well-studied Ca^2+^-triggered exocytosis models, the only identifiable domain in TgDOC2 is the namesake double C2 domain (“DOC2”), making its organization unconventional. In model organisms, Ca^2+^-mediated vesicle fusion with the plasma membrane is organized by at least three DOC2 domain proteins, of which at least one contains a transmembrane domain (15–18). C2 domains bind to other proteins or phospholipids. Ca^2+^ can make these C2 domain interactions conditional through its association with positionally conserved Asp residues in a select number of C2 domains (15, 19). The ferlins are unique among the DOC2 domain proteins as they contain five to seven C2 domains rather than two and are relatively large (∼200 kDa). The ferlins comprise an ancient eukaryotic protein family present in most unicellular organisms (except amoebas and fungi), including the Apicomplexa and all multicellular organisms (except higher plants) (20). Although ferlins are relatively understudied due to their absence from neurons and yeast, they typically function in membrane fusion, vesicle trafficking, and membrane repair. Dysfunction of human ferlins can cause deafness and muscular dystrophy (21).

To better understand the machinery underlying Toxoplasma Ca^2+^-mediated exocytosis, we evaluated the DOC2 domain family in the Apicomplexa. Next to the unconventional TgDOC2 (14), we identified three DOC2 proteins of the ferlin family, two of which are widely conserved across Apicomplexa. We determined that TgFER2, a conserved representative, is essential for host cell invasion and required for rhoptry secretion. These findings provide critical insight into the poorly understood mechanisms of rhoptry secretion while raising the possibility that, contrary to common assumptions, rhoptry secretion might be a Ca^2+^-dependent process.

## RESULTS

### The T. gondii genome encodes three ferlin proteins.

**FIG 1:**
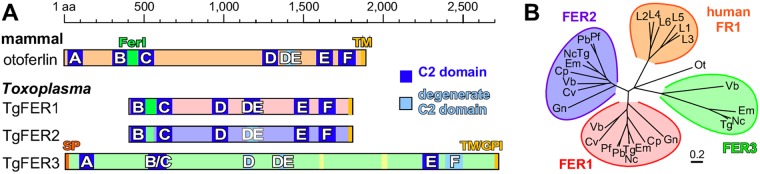
(A) Toxoplasma encodes three ferlin proteins, and human otoferlin is shown for comparison. Ferlins are defined by 5 to 7 C2 domains (labeled A to F) and a C-terminal transmembrane (TM) domain, as well as typically a “FerI” domain of unknown function. TgFER3 contains an N-terminal signal peptide, which, in combination with the C-terminal TM domain, could signal glycosylphosphatidylinositol (GPI) anchor addition at the C terminus. Yellow shading in TgFER3 represents coiled-coil domains. Degenerate C2 domains are defined as having a *P* value below the cutoff in Pfam database searches. (B) Phylogenetic analysis of apicomplexan, chromerid, and human ferlins. Abbreviations are grouped as follows. Human ferlins: L1 to L6, FR1L1 to -6 (FR1L1, dysferlin [O75923.1]; FR1L2, otoferlin [Q9HC10.3]; FR1L3, myoferlin [Q9NZM1.1]; FR1L4, A9Z1Z3.1; FR1L5, A0AVI2.2; FR1L6, Q2WGJ9.2). Ot, green algae Ostreococcus tauri (Q01FJ7). Chromerids: Vb, Vitrella brassicaformis (VbFER1, Vbre_12074 plus Vbra_12075; VbFER2, Vbra_9198); and Cv, Chromera velia (CvFER1, Cvel_17519.2; CvFER2, Cvel_9223). Apicomplexa: Tg, Toxoplasma gondii (TgFER1, TGME49_309420; TgFER2, TGME49_260470; TgFER3, TGME49_295472 plus TGME49_295468); Nc, Neospora caninum (NcFER1, NCLIV_053770; NcFER2, NCLIV_026570; NcFER3, NCLIV_002280); Em, Eimeria maxima (EmFER1, EMWEY_00002120; EmFER2, EMWEY_00009280; EmFER3, EMWEY_00017650); Pf, Plasmodium falciparum (PfFER1, PF3D7_0806300; PfFER2, PF3D7_1455600); Pb, Plasmodium berghei (PbFER1, PBANKA_122440; PbFER2, PBANKA_131930); Cp, Cryptosporidium parvum (CpFER1, cgd8_2910; CpFER2, cgd2_2320); and Gn, Gregarina niphandrodes (GnFER1, GNI_063830; GnFER2, GNI_073830). Alignment and the unrooted Jukes-Cantor phylogenetic tree were generated in Geneious (v.6.1.6) (62) from a MUSCLE alignment using neighbor joining. Note that the FER1 and FER2 nodes for T. gondii and N. caninum are barely discernible at this scale.

Next to TgDOC2, a series of BLAST searches of the Toxoplasma genome identified four additional proteins containing two or more C2 domains, of which three also contained a transmembrane domain. Two proteins had clear homology to the ferlin family of Ca^2+^-sensitive membrane fusion proteins (Fig. 1A). We named these proteins TgFER1 (TGME49_309420) and TgFER2 (TGME49_260470). The other DOC2 proteins, TGME49_295472 and TGME49_295468, are adjacent in the genome but the genes coding for them are annotated as a single gene in the ontological region in Neospora and Eimeria spp. This merged protein also possesses the global architecture of a ferlin and was named TgFER3. However, it diverges from the family by its extensive degeneration of C2 domains and its bigger size of 297 kDa (Fig. 1A).

Using human otoferlin as a reference, the C2 domains in T. gondii ferlins 1 to 3 follow the typical paired C2 pattern (Fig. 1A). The absence of the C2A domain in TgFER1 and TgFER2 is not unusual as this domain is missing in the majority of studied ferlins (20). All ferlins studied to date contain the FerI domain of as yet unknown function. This domain is present in TgFER1, slightly degenerate in TgFER2, and undetectable in TgFER3. We queried the conservation of ferlins in representative apicomplexan organisms and their closest free-living relatives, the Chromerids (22). Clear orthologs of TgFER1 and TgFER2 were universally present, but TgFER3 orthologs were restricted to the Coccidia (Neospora, Sarcocystis, and Eimeria) and, somewhat surprisingly, to the chromerid Vitrella brassicaformis (Fig. 1B). This suggests that TgFER3 was present in the last common ancestor of Chromerids and Apicomplexa but was lost from all apicomplexan lineages except the Coccidia. Overall, the Toxoplasma genome harbors four DOC2 family proteins: our previously reported TgDOC2 and FER1 to -3.

### TgFER2 is essential for completing the lytic cycle.

**FIG 2:**
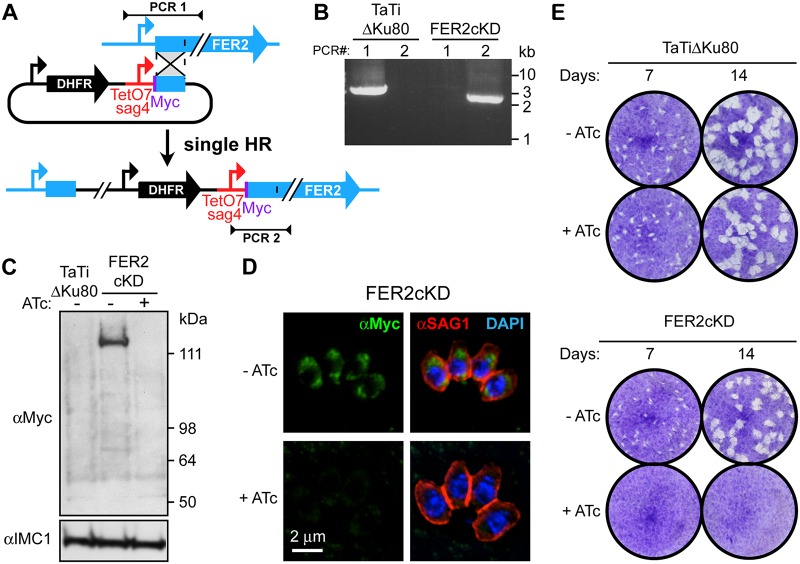
Generation and validation of a TgFER2 conditional knockdown (cKD) parasite. (A) Schematic representation of single homologous promoter replacement with the anhydrous tetracycline (ATc)-regulatable promoter TetO7sag4. Note that a Myc epitope tag is simultaneously added on the N terminus. Sites of diagnostic primer pairs used in panel B are indicated. (B) Diagnostic PCR of the parent line (TaTiΔKu80) and the FER2-cKD promoter replacement line using the primer pairs depicted in panel A. (C) Western blot demonstrating the conditional expression of the Myc-tagged TgFER2 allele. TgFER2 is downregulated to undetectable levels after 48 h of ATc treatment. Anti-IMC1 is used as a loading control. (D) Immunofluorescence demonstrating the loss of Myc-TgFER2 expression upon ATc treatment for 20 h. Parasites were fixed with 100% methanol. DAPI (4′,6-diamidino-2-phenylindole) labels DNA, and anti-SAG1 marks the plasma membrane. (E) Plaque assays of the parent (TaTiΔKu80) and FER2-cKD lines ± ATc treatment for the times indicated. No plaques are observed upon loss of TgFER2 expression.

Given the documented roles of DOC2 and ferlins in Ca^2+^-mediated secretion, we hypothesized that apicomplexan ferlins are involved in microneme secretion. To test this hypothesis, we probed the function of TgFER2 by replacing its promoter with a tetracycline-regulatable promoter (23) and simultaneously inserted a single N-terminal Myc epitope to provide localization data (Fig. 2A and B). Western blots of total parasite lysates probed with anti-Myc antibodies marked a single protein consistent with the 160-kDa predicted molecular weight of TgFER2 (Fig. 2C). Regulation of TgFER2 was demonstrated by exposing FER2 conditional knockdown (Fer2-cKD) parasites to anhydrous tetracycline (ATc) for 48 h to block TgFER2 transcription. Myc-TgFER2 was undetectable by Western blotting (Fig. 2C) or immunofluorescence assay (IFA) (Fig. 2D), confirming efficient protein knockdown. TgFER2-depleted parasites did not form plaques after 7 or 14 days (Fig. 2E). No observable changes in the morphology or growth rate of intracellularly replicating parasites were observed (see Fig. S1A in the supplemental material). TgFER2 therefore does not function in cell division or replication but is essential for completion of Toxoplasma’s lytic cycle.

10.1128/mBio.01510-18.1 FIG S1 Phenotypic characterization of FER2-cKD parasites. (A) Replication assessment of FER2-cKD parasites. Following 24 h of preincubation ± ATc, parasites were allowed to replicate in host cells for 24 h ± ATc, at which point the number of parasites per vacuole was counted (48 h of ATc treatment total). At least 100 parasites per condition as indicated were counted. *n* = 1. (B) TgFER2-depleted parasites do not display an egress defect. Treatment with 2 μM Ca^2+^ ionophore A23187 for 5 min induced egress. The number of egressed versus nonegressed vacuoles was counted. At least 100 vacuoles were counted. Induction with 1 μg/ml ATc marked with “+” reflects 48 h and “++” reflects 96 h. *n* = 3 + SD. (C) TgFER2-depleted parasites do not display a conoid extrusion defect. Parasites were treated ± ATc for 48 h, conoid extrusion was induced with 0.5 M ethanol for 30 s, parasites were fixed with 1.25% glutaraldehyde, and extruded conoids were counted blind by microscopy. At least 350 parasites were counted (*n* = 2). Both collected data points are shown; bars represent average. Download FIG S1, TIF file, 0.09 MB. Copyright © 2018 Coleman et al. 2018 Coleman et al. This content is distributed under the terms of the Creative Commons Attribution 4.0 International license.

### TgFER2 does not localize to the micronemes.

Myc-TgFER2 localization by IFA revealed a dispersed pattern not reminiscent of any Toxoplasma feature (Fig. 2D). Since membrane trafficking proteins are notoriously hard to accurately localize (e.g., synaptotagmin IV is observed on different membranes in the secretory pathway, dependent on cell type, condition, and fixation method [24–26]), we tested fixatives like paraformaldehyde, methanol, and acetone. Although the control SAG1 pattern varied per fixative, the Myc pattern remained unchanged (see Fig. S2A in the supplemental material). Because it is conceivable that TgFER2 localization changes upon egress and/or in response to a change in [Ca^2+^]_i_, we also probed extracellular parasites ± treatment with the Ca^2+^ ionophore A23187. Under any extracellular condition, TgFER2 appears as defined cytoplasmic puncta suggestive of either inclusion bodies or a membranous structure of unknown identity (see Fig. S2B). This staining pattern differed from the more disperse intracellular signal, which is likely a result of the different physiological states (e.g., [Ca^2+^]_i_) of the parasites under these conditions.

10.1128/mBio.01510-18.2 FIG S2 IFA localization of Myc-FER2 under various conditions. (A) Parasites expressing an N-terminally tagged TgFER2 from the endogenous locus under the Tet7sag4 promoter fixed with 100% methanol (MetOH), 4% paraformaldehyde (PFA), or 100% acetone costained with SAG1 antiserum to mark the plasma membrane. Fixative choice does not affect the localization pattern observed for Myc-TgFER2, but does affect the appearance of SAG1. (B) Extracellular parasites treated with 5 μM Ca^2+^ ionophore A23187 and vehicle control for 5 min in Ringer’s solution were fixed with 100% methanol and stained with Myc and SAG1 antisera. Note the extended conoid in the A23187-treated condition indicative of a Ca^2+^-mediated response. Regardless of ionophore treatment, the Myc-FER2 signal morphed into a punctate pattern spread throughout the cytoplasm compared to the intracellular condition. The punctate pattern does not reflect any known organelle or trafficking pathway in Toxoplasma and might suggest aggregation of the protein. Download FIG S2, TIF file, 3.38 MB. Copyright © 2018 Coleman et al. 2018 Coleman et al. This content is distributed under the terms of the Creative Commons Attribution 4.0 International license.

**FIG 3:**
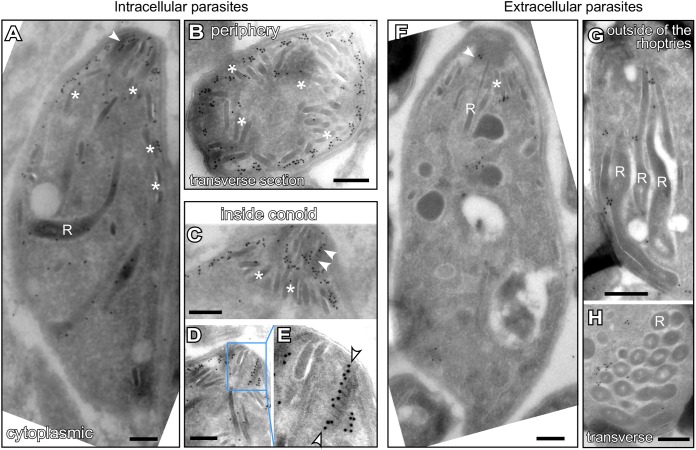
Subcellular localization of TgFER2. Shown is immunoelectron microscopy of intracellular (A to D) and extracellular (F to H) Toxoplasma tachyzoites expressing an N-terminal Myc epitope-tagged TgFER2 from the endogenous locus under the TetO7sag4 promoter. In intracellular parasites, Myc antibodies direct gold particle clusters to the cytoplasmic side of the IMC, and there is a strong enrichment inside the conoid, but no strong association with micronemes and minor association with the rhoptries. In extracellular parasites, gold particles are predominantly observed in clusters on the cytoplasmic side of the rhoptry membranes next to localization inside the conoid. (Note the experimental variation: Myc antibody binding was not as efficient in extracellular parasites.) “R” marks the rhoptries, asterisks mark micronemes, and arrowheads mark gold beads in the conoid. Panel E is a magnification of the region marked in panel D. Scale bars are 250 nm.

Since the transmembrane domain is predictive of membrane association, we resolved the localization by immunoelectron microscopy (IEM). In intracellular parasites, a comparatively small number of gold particles were distributed throughout the cytoplasm (Fig. 3A). Gold particles were notably enriched at the cytoplasmic side of the inner membrane complex (IMC) (Fig. 3A and B) and within the internal structures of the conoid at the apical end (Fig. 3A and C to E). In extracellular parasites, we observed a different pattern with gold particles patched on the cytoplasmic side of the rhoptries (Fig. 3F to H). The lack of consensus across multiple IFA and IEM experiments and the technical limitations of both methods restrain us from drawing any definitive conclusion about FER2 localization in the tachyzoites. Nonetheless, TgFER2 labeling was never observed on the micronemes, which was inconsistent with our initially hypothesized role for FER2 in microneme secretion.

### TgFER2 is not required for microneme secretion and conoid extrusion.

To unravel the lethality of TgFER2 depletion, we first assayed parasite egress. After 48 or 96 h of ATc treatment, FER2-cKD parasites egressed normally when treated with Ca^2+^ ionophore A23187 (Fig. S1B). This suggests that the micronemes are secreted normally. The morphology and distribution of micronemes in TgFER2-depleted parasites are also normal by IFA and transmission electron microscopy (TEM) (see Fig. S3 in the supplemental material). Since TgFER2 was detected in the conoid, we also examined conoid extrusion as another Ca^2+^-regulated process (27). Figure S1C in the supplemental material shows that conoid extrusion is normal in the TgFER2-depleted mutant.

10.1128/mBio.01510-18.3 FIG S3 Depletion of TgFER2 does not affect microneme formation, morphology, or number. (A) IFA of extracellular FER2-cKD parasites for two different microneme proteins, AMA1 and Mic2, shows that the micronemes localize to the correct apical location. DAPI stains DNA. (B to E) Transmission electron microscopy of parasites induced ± ATc for 48 h, incubated for 6 h with host cells, and then fixed. Apical ends are positioned at the top of the images. (B) Completely invaded TgFER2-replete parasite residing in a vacuole displaying a released rhoptry. (C) TgFER2-depleted parasite partly engulfed by a host cell displaying rhoptries and micronemes in the apical end. “N” marks the nucleus. The box is enlarged in the bottom left inset illustrating the absence of a MJ at the host-parasite interface. (D) Extracellular, FER2-depleted parasites displaying rhoptries and micronemes present in the apical end. Note that of the four parasite cross sections shown, all show many micronemes at the apical end, except for the parasite at the center right, which displays very few. (E) Counts of the number of micronemes per parasite as indicated. Only parasites with longitudinal sections through the entire parasite were included in the analysis. Individual data points are shown; each horizontal red line represents the average, and error bars denote SD. Download FIG S3, TIF file, 2.88 MB. Copyright © 2018 Coleman et al. 2018 Coleman et al. This content is distributed under the terms of the Creative Commons Attribution 4.0 International license.

**FIG 4:**
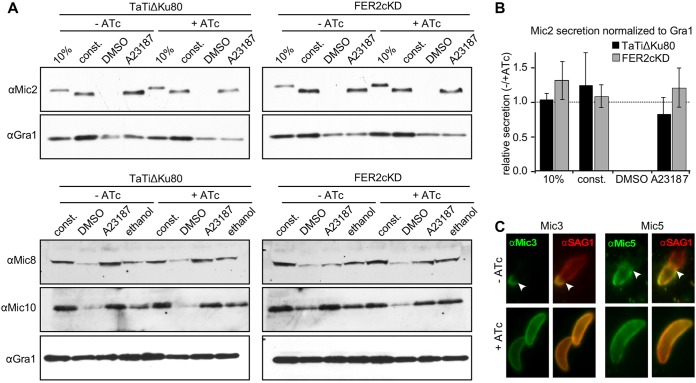
Microneme secretion of TgFER2-depleted parasites. (A) Microneme secretion assay by Western blotting. The lane labeled “10%” shows total parasite lysate corresponding with 10% of the parasites used in the secretion assay; “const.” represents constitutive secretion of extracellular parasites for 1 h; “A23187” and “DMSO” represent induced secretion with Ca^2+^ ionophore (1 μM A23187) and the vehicle control for 5 min. “Ethanol” represents 1% ethanol as a trigger for microneme secretion. Microneme secretion of the classic population is detected by Western blotting with anti-Mic2, which shows a size shift upon secretion, and with anti-Mic10. Secretion of the Mic3/5/8/11 microneme population is monitored with anti-Mic8. Anti-Gra1, which detects dense granule secretion, is used as control. (B) Quantitation of Mic2 secretion normalized to GRA1 secretion shown in panel A. *n* = 3 ± standard deviation (SD). No statistically significant differences were detected. (C) Secretion of the Mic3/5/8/11 population monitored by IFA using anti-Mic3 and anti-Mic5 (anti-Mic8 data in Fig. S4A and B in the supplemental material). Extracellular FER2-cKD parasites ± ATc were placed on HFF cells. Host cells were permeabilized by 0.02% saponin so that only secreted Mic is detected (parasites are not permeabilized under this condition). Anti-SAG1 marks the plasma membrane. An arrowhead marks the site of invading parasites at the boundary where the apical end of parasites is already inside the host cell and stripped of nearly all Mic and most SAG1 protein. Single-color and phase panels are shown in Fig. S4.

Next we directly tested microneme protein secretion through Mic2 release (28). Both untriggered, low-level constitutive secretion and Ca^2+^ ionophore-induced microneme secretion occurred normally in the absence of TgFER2 (Fig. 4A and B). It is now apparent that micronemes are not uniform and that distinct populations within the parasite contain different proteins (29). We therefore reasoned that TgFER2 might act differentially on these populations and that this might explain our observations. Mic2 is secreted from a Rab5a/c-dependent population of micronemes. Another component of this population, Mic10, was also secreted normally in TgFER2-depleted parasites (Fig. 4A). We examined secretion from the Rab5a/c-independent population Mic proteins 3, 5, 8, and 11 containing population by assessing Mic8 secretion by Western blotting. This proceeded normally in the absence of TgFER2. Labeling of Mic3, -5, and -8 by IFA on nonpermeabilized parasites (30, 31) also confirmed secretion of these proteins to the surface of both FER2-replete and -depleted parasites (Fig. 4C; see Fig. S4A and B in the supplemental material). Thus, secretion of all micronemes is TgFER2 independent.

10.1128/mBio.01510-18.4 FIG S4 Mic3, Mic5, and Mic8 secretion and motility are normal upon TgFER2 depletion. (A and B) Arrowheads mark the site of host cell invasion. Note the gliding motility trails in the lower panels of both panels A (−ATc) and B (+ATc). (C) Quantification of motility modes for FER2-cKD parasites ± ATc for 96 h. Motility of parasites on 50% FBS-coated glass slides was observed for 90 s by video microscopy and scored for the type of motility. Data are expressed as the percentage of the observed motility mode of the total number of observed parasites (*n* as indicated at bottom of the graph). Download FIG S4, TIF file, 2.03 MB. Copyright © 2018 Coleman et al. 2018 Coleman et al. This content is distributed under the terms of the Creative Commons Attribution 4.0 International license.

Surface antigen SAG1 and Mic8 were deposited in trails behind parasites ± ATc, implying that TgFER2-depleted parasites are still motile (Fig. S4A and B). This was confirmed by scoring the total number of motile parasites and the type of motility displayed by individual FER2-cKD parasites by video microscopy (Fig. S4C). However, invading parasites, which are clearly identifiable by the stripping of Mic proteins off the apical invading parasite surface, were only observed in the presence of TgFER2 (Fig. 4C; Fig. S4A), suggesting a microneme-independent invasion defect.

### TgFER2 is required for host cell invasion.

We further examined host cell invasion through a series of invasion and attachment assays. As controls, we used mutants with defects at different points of host cell attachment and/or invasion. These include the TgDOC2 temperature-sensitive mutant (*ts-*DOC2) devoid of all microneme secretion (14), the calcineurin (CnA) mutant, which secretes micronemes normally but does not attach properly (9), the AMA1 mutant, which secretes micronemes but shows an increase in aborted invasion events due to failures in functional MJ formation (32, 33), and the DHHC7 mutant, which lacks the palmitoyltransferase responsible for anchoring the rhoptries at the apical end and as a result is defective in rhoptry secretion (5).

**FIG 5:**
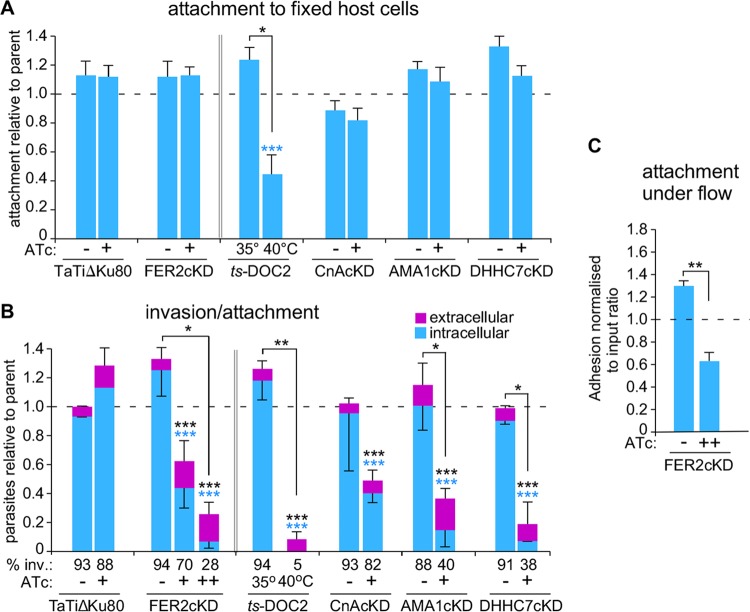
Invasion and attachment of TgFER2-depleted parasites. (A) Attachment to fixed HFF cells. Equal numbers of mutant parasites across the experiments were mixed 1:1 with the internal control (TaTiΔKu80 parasites expressing cytoplasmic YFP) and exposed to fixed HFF cells. All parasites were stained with anti-SAG1, and control versus test parasites were counted. Data are expressed relative to the internal control of TaTiΔKu80 minus ATc (for *ts*-DOC2 TaTiΔKu80 was used for comparison rather than its direct parent). Mutants were induced with ATc for 48 h, except *ts*-DOC2, which was induced at 40°C for 48 h. The dotted line represents the internal control level. *n* = 5 + standard error (SE). Across-samples statistics for ±ATc: ***, *P*  < 0.0001 by one-way ANOVA. Pairwise statistics for ±ATc: *, *P*  = 0.014 by Student’s *t* test. (B) Red-green invasion assay to determine invasion and attachment efficiency using a constant number of parasites across the various mutants and controls. Extracellular parasites were differentially stained from intracellular parasites with Alexa 488-conjugated anti-SAG1 before fixation; all parasites were subsequently stained following fixation and permeabilization with Alexa 594-conjugated anti-SAG1. For FER2, the presence of ATc marked with “+” represents 48 h and “++” represents 96 h. *n* = 3 ± SD. Across-samples statistics for ±ATc (one-way ANOVA): colored asterisks represent the variable compared across samples, and black asterisks represent the total number of parasites. The percentage of invaded parasites is indicated at the bottom. Pairwise statistics for ±ATc (Student's *t* test): *, *P*  < 0.01, **, *P*  = 0.001, and ***, *P*  < 0.0001. (C) Parasite attachment to HUVECs under fluidic shear stress. Levels of adhesion of the FER2-cKD line ± ATc (96 h) were compared. Parasite adhesion normalized to the ratio of each parasite population introduced into the fluidic channel is shown, wherein a value of 1.0 represents equivalent adhesion of the two populations. *n* = 3 + SD. **, *P*  < 0.01 (Student's *t* test). See Fig. S5 for additional controls.

Early events in parasite attachment are mediated by the binding of SAG proteins to glycans on the host cell surface. We assayed this by attachment of parasites to fixed host cells (30). Only the *ts*-DOC2 mutant, which completely fails to secrete micronemes, demonstrated reduced attachment relative to the wild-type and uninduced controls. Thus, all other mutants have normal microneme secretion (Fig. 5A). These data in general indicated that micronemal proteins support initial attachment, in the absence of MJ formation as that is not possible on fixed host cells. Next we tested attachment and invasion by the “red-green invasion assay” (34). TgFER2-depleted parasites invaded at a much lower frequency (Fig. 5B). The defect intensified >3-fold upon prolonged TgFER2 depletion (96 h), which importantly did not affect their viability as these parasites are still fully capable of egress (Fig. S1B). As expected, all other mutants showed severe invasion defects. In this assay, quantification of the total number of parasites per field allows for an estimation of parasite attachment. By this metric, a defect in the attachment of TgFER2-depleted parasites to host cells was observed. By 96 h of knockdown, the numbers of parasites attached to host cells dropped nearly 4-fold (Fig. 5B). This approaches the levels observed in the *ts*-DOC2 mutant, where attachment and invasion are both severely defective. The similarity between the *ts*-DOC2 mutant, where the primary defect is in attachment, and the DHHC7 mutant, with a penetration defect, highlights that this assay is unable to distinguish between these two interconnected phenotypes.

10.1128/mBio.01510-18.5 FIG S5 Controls for parasite adhesion to HUVECs under fluidic shear stress. Adhesion of each parasite line normalized to the input ratio of the parasites introduced into the channel is shown. A value of 1.0 represents equivalent adhesion of the two populations. Three independent fluidic experiments were performed comparing these two lines, and the combined data are shown. ***, *P*  < 0.001 (Student’s *t* test). Download FIG S5, TIF file, 0.04 MB. Copyright © 2018 Coleman et al. 2018 Coleman et al. This content is distributed under the terms of the Creative Commons Attribution 4.0 International license.

**FIG 6:**
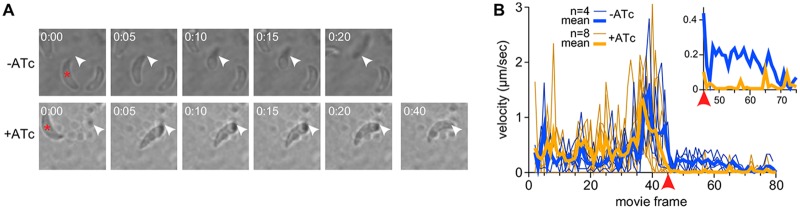
Impulse motility and host cell invasion. (A) Still panels from movies collected in Movie S1 recorded with FER2-cKD parasites ± ATc in the presence of host cells. The TgFER2-replete parasite marked with the asterisk invades the host cell at the arrowhead. Invasion is complete in 20 s. The TgFER2-depleted parasite marked with asterisk makes an impulse move to the arrowhead and appears to deform the host cell. However, the parasite does not invade and disengages from the host cell, reversing the deformation in the 40-s frame. (B) Velocity profiles of FER2-cKD parasites ± ATc. The red arrowhead marks the synchronized frame where the parasites minus ATc invade or the parasites plus ATc engage the host cell. Each thin line represents a single parasite from a single movie; heavy lines represent mean values for all parasites in each group included in the graph. Note that both sets of parasites show an impulse in motility right before the point of invasion/engagement, followed by an immediate pause, but that only the TgFER2-replete parasites maintain a positive velocity during the actual host cell invasion (magnified in the inset).

It has been observed that the motility and attachment dynamics of Toxoplasma are different under conditions of shear stress (35). To investigate whether these conditions might better clarify the phenotype of the TgFER2 knockdown, we measured the ability of FER2-cKD parasites ± ATc to adhere to human vascular endothelial cells (HUVECs) under flow. Depletion of TgFER2 led to a significant decrease in the number of parasites retained in the chamber (Fig. 5C; see Fig. S5 in the supplemental material), although attachment of TgFER2-depleted parasites was less compromised under flow relative to static conditions. This further underscores that TgFER2 is essential for invasion but does not pinpoint the nature of the defect. FER2-cKD parasites were then scrutinized for their interactions with host cells by video microscopy. Both wild-type and FER2-cKD parasites were able to glide across the host cells. In contrast to control parasites, TgFER2-depleted parasites were not able to invade host cells (Fig. 6; see Movie S1 in the supplemental material). Surprisingly, FER2-cKD parasites still exhibited “impulse motility” characteristic of invading parasites (36). This typical burst of forward motion immediately preceding invasion is followed by a momentary pause when parasites secrete the RONs and create the MJ before proceeding with invasion and subsequent parasitophorous vacuole formation. Both control and induced FER2-cKD parasites displayed bursts of impulse motility followed by a pause. However, only in control parasites was this pause followed by forward motion (invasion) at approximately half the original speed. In contrast, the velocity of induced parasites dropped essentially to 0 μm/s, and they failed to invade. This observation suggests that TgFER2 functions in the very late stages of invasion and indicates that the MJ either is not formed or is not of sufficient strength to support the force required for parasite penetration into cells.

10.1128/mBio.01510-18.8 MOVIE S1 Impulse motility and host cell invasion. Combined movie of all 12 FER2-cKD parasites analyzed for Fig. 6 synchronized at the point of host cell invasion (−ATc) or engagement (+ATc). Time-lapse movies were collected (and are shown) for different times at a rate of 15 frames/s. Download Movie S1, AVI file, 5.62 MB. Copyright © 2018 Coleman et al. 2018 Coleman et al. This content is distributed under the terms of the Creative Commons Attribution 4.0 International license.

### TgFER2 is required for rhoptry secretion.

**FIG 7:**
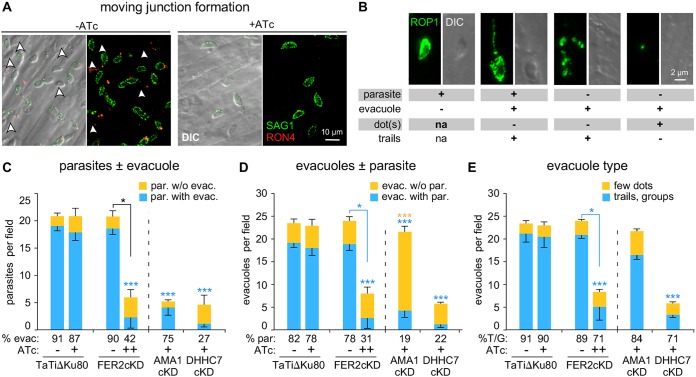
Rhoptry secretion of TgFER2-depleted parasites is impaired. (A) Formation of the MJ. A constant number of parasites across the various mutants and controls were incubated with host cells for 10 min. MJ formation was visualized with RON4 antiserum under semipermeabilizing conditions by 0.02% saponin. SAG1 stains the extracellular portion of the parasites. Arrowheads mark successfully invaded parasites that are not accessible to the SAG1 antibodies. Brightness and contrast adjustments were made identical for both conditions, and, thus, signals are directly comparable. (B) Representative examples of parasite and evacuole features scored in the evacuole assay represented in panels C to E. na, not applicable. (C to E) Evacuole assay to monitor rhoptry bulb secretion and assess stability of the MJ attachment. Parasites as indicated were grown under ATc for 48 h (+) or 96 h (++) and incubated with host cells for 10 min. Evacuole formation was visualized using ROP1 antiserum following paraformaldehyde fixation. *n* = 3 ± SD. For across-samples statistics for ±ATc (one-way ANOVA), correction is marked above the bar. Pairwise statistics for ±ATc (Student’s *t* test) are marked above the connector line. *, *P*  < 0.01; **, *P*  = 0.001; ***, *P*  < 0.0001. Asterisk color corresponds with the variable compared across samples; black asterisks correspond with analysis of the total number of parasites. Indicated below the graphs in panels C, D, and E are the percentages of the total number of events recorded that represent association with the evacuole (evac.) or parasite (par.) or display trails/groups of evacuoles, respectively. The data presented in these panels are derived from the same experiments.

As shown in Fig. 3 and Fig. S3 and S6 in the supplemental material, the localization, morphology, and apical anchoring of the rhoptries were not affected by TgFER2 depletion. Thus, FER2 is not required to tether the rhoptries at the apical end of the parasite. To test rhoptry function, we first monitored the release of rhoptry neck proteins by tracking RON4 distribution and assaying MJ formation (Fig. 7A). In the noninduced control, we readily observed MJ formation, but no MJ formation was detected in the absence of TgFER2. These data suggest that the RON proteins are either not secreted, or if they are, they fail to assemble into the MJ.

10.1128/mBio.01510-18.6 FIG S6 Depletion of TgFER2 does not affect rhoptry formation, morphology, or anchoring. Shown is IFA demonstrating proteins correctly target to the rhoptries, which localize to the apical end of the parasite. Extracellular parasites are stained with ROP1 antiserum to mark the rhoptries, and IMC3 stains the cortical cytoskeleton outlining the parasites. DIC delineates the vacuoles. DAPI stains DNA. In the vacuoles containing parasites organized in rosettes, the apical ends are facing outward. DHHC7-cKD parasites, wherein rhoptry anchoring at the apical end is disrupted (5), are shown as a control and display ROP1 puncti randomly organized in the cytoplasm. Download FIG S6, TIF file, 5.18 MB. Copyright © 2018 Coleman et al. 2018 Coleman et al. This content is distributed under the terms of the Creative Commons Attribution 4.0 International license.

We next performed “evacuole” assays to determine the quantity and quality of rhoptry protein secretion in TgFER2 mutants. Evacuoles, which are rhoptry protein clusters injected in the host cell cytosol, were visualized with ROP1 antiserum and classified as illustrated in Fig. 7B (33). We used the AMA1-cKD as control for partial rhoptry secretion and weak MJ strength (32, 33) and DHHC7-cKD parasites as a control for defective rhoptry secretion (5). To examine the strength of the overall parasite-host cell interaction, the number of parasites per field was counted and differentiated by whether they were associated with evacuoles (Fig. 7C). More specifically, the strength of the MJ itself can be measured by assaying the number of evacuoles per field and whether they are associated with parasites (Fig. 7D). Levels of rhoptry secretion were differentiated by the relative size of evacuole patterns (Fig. 7E): small punctate ROP1 staining indicates less secretion than long trails or clusters. For FER2-cKD parasites, the number of parasites per field was consistent with the observations from the attachment and invasion assays: depletion of TgFER2 decreased parasite attachment (Fig. 7C). Among the parasites that were attached, very few were associated with evacuoles, indicating that they have not secreted the rhoptries’ contents into the host cell. The TgFER2 data are comparable with the results for the DHHC7 mutant. However, they differed from parasites that lack AMA1, where few parasites attach but the majority of the parasites have secreted rhoptries and generated evacuoles (32, 33). TgFER2-depleted parasites, like the DHHC7 mutant, appear to secrete very few, if any, rhoptries. When we assess how many of the observed evacuoles are associated with parasites, it becomes clear that TgFER2 depletion is much more similar to DHHC7 depletion than to parasites lacking AMA1 (Fig. 7D). Finally, we observed relatively few extensive evacuole patterns for both TgFER2- and DHHC7-depleted parasites (Fig. 7E) and conclude that in the rare events of rhoptry secretion, very little material was released. Overall, we conclude that TgFER2 is required for the secretion of the rhoptries, which is necessary to invade host cells.

## DISCUSSION

Micronemes and rhoptries are essential to the invasion of apicomplexan parasites. These fascinating cellular structures are likely derived from ancestral organelles that persist in modern predatory protozoa and were adapted during the evolution of the Apicomplexa’s intracellular, parasitic lifestyle (37). While the molecular details of the initiation of microneme secretion are incompletely understood, the critical role of intracellular Ca^2+^ fluxes has been known for decades. Far less is known about either the mechanisms of rhoptry secretion or the trafficking of their contents.

It has long been hypothesized that secretion from both micronemes and/or rhoptries requires a membrane fusion event, but evidence for a canonical secretion machinery has been elusive. Using C2 domains as the anchor for a bioinformatic search for potential components of this pathway, we were unable to find homologs for either synaptotagmins or the canonical DOC2 family of Ca^2+^ sensors that function in mammalian neurotransmitter release (18, 38). We did find orthologs of the ferlin family of Ca^2+^-sensing membrane fusion proteins. TgFER1 and TgFER2 are widely conserved across the Apicomplexa, whereas the degenerate TgFER3 is found only in the Coccidia and a single Chromerid species, illustrative of the ancient history of these processes.

Detailed studies of Toxoplasma FER2 demonstrated its requirement for secretion from the rhoptries. This finding provides one of the first mechanistic insights into rhoptry secretion, firmly linking it to the activity of this C2 domain-containing protein. It is generally accepted that rhoptry secretion must be preceded by microneme secretion and requires contact with an appropriate host cell (4, 39). However, neither the transduction of this attachment signal nor the process by which it leads to secretion of the organelle’s contents has been clarified. Although it is known that Mic8 is required for rhoptry release and has been postulated to be key in a signal transduction pathway (40), there are no experimental data supporting this model. Furthermore, AMA1 (33) and RON5 (41) also appear to be involved in rhoptry secretion, but these mechanisms are equally unknown. Our finding that the Ca^2+^ sensor TgFER2 is required for rhoptry secretion provides a tantalizing hint at the molecular mechanism. The presence of an Asp residues constellation consistent with Ca^2+^-binding capacity in TgFER2’s C2F domain supports this model (see Fig. S7 in the supplemental material). This is consistent with Ca^2+^ binding being restricted to the C2E and C2F domains in mammalian ferlins (21). We have as yet been unable to definitively demonstrate a role for Ca^2+^ in TgFER2 function specifically. However, ferlins are Ca^2+^-sensing proteins, and it is well established that a rise in [Ca^2+^]_i_ accompanies host cell invasion (2). Thus, while the conventional belief has been that this fluctuation acted only on activation of motility, conoid extrusion, and microneme secretion, our collective evidence provides a hint that rhoptry secretion may be similarly dependent on variations in [Ca^2+^]_i_. The relative importance of the protein’s individual C2 domains in this process and their relative Ca^2+^-binding abilities are exciting open questions to be experimentally determined.

10.1128/mBio.01510-18.7 FIG S7 Modeling of Ca^2+^-binding capacity in the C2 domains for TgFER2. The insights and alignments described by Jiménez and Bashir (63) were used to identify the 5 positions in the C2 domains of TgFER2 potentially interfacing with Ca^2+^. (A) Overview of C2 domains B to F and the direct surrounding of the amino acids in the 5 positions orthologous to Ca^2+^-binding amino acids in other ferlin C2 domains. The last column interprets the conservation data, taking into account the listed assumptions and the fact that positions 2, 3, and 4 are most critical to the ability to bind calcium. (B) Schematic of the amino acid sequence in the three loops of the TgFER2 C2F domain interfacing with Ca^2+^. Positions 1 to 5 are indicated. It is of note that some C2 domains can bind two Ca^2+^ ions, but C2F most likely can only accommodate one ion. Download FIG S7, TIF file, 0.18 MB. Copyright © 2018 Coleman et al. 2018 Coleman et al. This content is distributed under the terms of the Creative Commons Attribution 4.0 International license.

Of the mammalian ferlins, otoferlin is currently the best studied, yet its mechanism of action remains poorly understood (42). Otoferlin is expressed in many tissues, but in cochlear hair cells (CHCs) it controls the release of neurotransmitter upon an increase in [Ca^2+^]_i_ (43, 44). A rise in [Ca^2+^]_i_ leads otoferlin to interact with phospholipids (42) and SNARE proteins *in vitro* (45), although SNAREs have been debated to be absent from the site of secretion in CHCs (46). This highlights the potential for ferlin proteins to facilitate membrane fusion in the absence of SNAREs, an important parallel to Toxoplasma, in which there is currently no evidence for either rhoptry- or microneme-resident SNAREs.

As part of this study, we compared different invasion and egress mutants across several commonly used assays, which allowed for several important observations. First, the fixed host cell attachment data (MJ absent) demonstrate that microneme proteins contribute a large portion to the attachment strength. Somewhat unexpectedly, the red-green invasion assay did not differentiate the various mutants very well, with the exception of the partial attachment defect previously reported for the CnA mutant (9). Thus, this assay is not capable of specifically attributing individual phenotypes to defects in attachment versus invasion. In contrast, the evacuole assay was very powerful in differentiating different aspects of MJ formation and rhoptry secretion.

Overall, our findings support two interesting hypotheses. First, if ferlins act as Ca^2+^ sensors during Ca^2+^-dependent secretion in the Apicomplexa, TgFER2 may represent the link between the previously observed Ca^2+^ fluctuations during invasion and the well-described mechanics of MJ formation. If on the other hand, the essential role of TgFER2 during rhoptry secretion is calcium independent, this would signify a fascinating evolutionary divergence from the canonical function of ferlins as Ca^2+^ sensors. While additional work will be required to distinguish between these models, the work presented here is a critical step in our understanding of these critical virulence processes.

## MATERIALS AND METHODS

### Parasites and mammalian cell lines.

Transgenic derivatives of the RH strain were maintained in human foreskin fibroblasts (HFFs) as previously described (47). For the attachment assay under fluidic shear stress, HUVECs were cultured in EGM-2 medium containing EGM-2 SingleQuot supplements and growth factors (Lonza, Allendale, NJ). TgFER2 CDS was amplified using primers YFP-FER2-F/R and NheI/EcoRV cloned into tub-YFPYFP(MCS)/sagCAT (48) to generate ptub-YFP-FER2/sagCAT, which was used for Sanger sequencing validation of the gene model. FER2-cKD parasites were generated by BglII/NotI cloning of PCR-amplified FER2 sequence (primers BamHI-FER2-F/NotI-FER2-R) into N-terminal Myc epitope-tagged plasmid derived from dihydrofolate reductase (DHFR)-TetO7sag4-Nt-GOI (Wassim Daher, Université de Montpellier) and linearized by XbaI prior to transfection. *ts*-DOC2 parasites were generated by first 5xTY tagging the DOC2 locus using the PCR amplicon (primers 5xTy_upstream_F/5xTy_PlusLink_R) from plasmid pLIC-5xTY-DD24/HX (Chris Tonkin, Walter and Eliza Hall Institute) and BglII/EcoRV cloning into tub-YFPYFP(MCS)/sagCAT. The tub promoter was PmeI/BglII replaced with the 3′ DOC2 homologous region PCR amplified from genomic DNA (gDNA [primers DOC2_3-target_F/R]). The CAT cassette was PmeI/NotI replaced with a DHFR minigene cassette and plasmid NheI linearized prior to transfection. A CRISPR/Cas9 (clustered regularly interspaced short palindromic repeats with Cas9) plasmid was generated to mutate DOC2 F124 to S124 using primers DOC2_proto_F/R (49) and cotransfected with hybridized oligonucleotides DOC2_FM>SV_F/R in RHΔKu80ΔHX-DOC2-5xTY parasites. All primer sequences are provided in Table S1 in the supplemental material.

10.1128/mBio.01510-18.9 TABLE S1 Primers used in this study. Restriction enzyme sites are underlined. Download Table S1, DOCX file, 0.10 MB. Copyright © 2018 Coleman et al. 2018 Coleman et al. This content is distributed under the terms of the Creative Commons Attribution 4.0 International license.

### Imaging.

The following antisera were used: anti-Myc monoclonal antibody (MAb) 9E10, anti-SAG1 MAb DG52 (50), anti-Mic2 MAb 6D10 (51), mouse anti-AMA1 (33), rabbit anti-Mic3 (30), rabbit anti-Mic5 (52), rabbit anti-Mic8 (53), and mouse anti-ROP1 (54). Alexa 488- or 594-conjugated secondary antibodies were used (Invitrogen). Images were collected on a Zeiss Axiovert 200 M wide-field fluorescence microscope, and images were deconvolved and adjusted for phase contrast using Volocity software (Perkin Elmer).

### Egress assay.

The egress assay was performed as described previously (9, 14). Freshly lysed parasites, pretreated ± ATc for 24 h, were inoculated into HFF cells and incubated ± ATc for an additional 24 h. For 96 h, parasites treated ± ATc for 68 h were inoculated and incubated ± ATc for an additional 30 h. Egress was triggered by treatment with 2 μM A23187 or dimethyl sulfoxide (DMSO) at 37°C for 5 min, followed by IFA with rat anti-IMC3 (48). Intact vacuoles were counted for each sample in at least 10 fields, and the percentage of egress was calculated relative to the DMSO control.

### Attachment and invasion.

The combined attachment/invasion assay was performed as previously published (14, 34) with modifications described in reference 9. Tachyzoites treated ± ATc for the hour indicated (*ts*-DOC2 parasites incubated at 35 and 40°C) were added to host cells in a 96-well plate, centrifuged (28 × *g*, 3 min, room temperature), and allowed to invade for 1 h at 37°C. Noninvaded extracellular parasites were detected using Alexa 594-conjugated anti-SAG1 T41E5 (55). Following fixation and permeabilization, all parasites were visualized with Alexa 488-conjugated anti-SAG1 T41E5. At least 300 parasites were counted per sample.

### Attachment to fixed host cells.

Assay was performed as previously described (33). HFF confluent 96-well optical bottom plates were fixed with 3% formaldehyde (PFA) plus 0.06% glutaraldehyde for 5 min at 4°C, followed by overnight 0.16 M ethanolamine quenching at 4°C. Wells were prerinsed with 0.2% bovine serum albumin (BSA) in Dulbecco’s modified Eagle’s medium (DMEM). Cytoplasmic yellow fluorescent protein (YFP)-expressing TATiΔKu80 parasites mixed in a 1:1 ratio were used as an internal control (9), centrifuged (28 × *g*, 5 min, 20°C) on the monolayer, and incubated for 30 min at 37°C. Wells were rinsed 3 times with phosphate-buffered saline (PBS), fixed with 4% PFA for 30 min at 4°C, and permeabilized with 0.25% Triton X-100 for 10 min. After blocking with 1% BSA in PBS, the parasites were probed with rabbit anti-green fluorescent protein (anti-GFP [Torrey Pines Biolabs]), and mouse anti-SAG1 DG52. Three random fields in 3 independent wells were counted.

### Attachment under fluidic shear stress.

Attachment under fluidic shear stress was performed as described previously (35, 56). Microfluidic channels containing fibronectin were coated overnight with HUVECs. Freshly lysed parasites treated ± ATc for 48 or 96 h were either stained with CMTPX CellTracker red or carboxyfluorescein succinimidyl ester (CFSE [Life Technologies]), counted, and combined 1:1. In each replicate experiment, the dyes were switched on the parental and knockdown parasite lines. Parasites were flowed at a shear force of 0.5 dyne/cm^2^ for 10 min at 37°C and were fixed under flow conditions with 4% PFA for 30 min, followed by imaging on a Nikon Eclipse Ti microscope.

### Conoid extrusion assay.

The conoid extrusion assay was performed as published (27). Freshly lysed parasites grown ± ATc for 48 h were resuspended in 10% fetal bovine serum (FBS) in HS buffer. Conoid extrusion was induced using 0.5 M ethanol or 5 µM A23187 for 30 s. Parasites were fixed and scored for conoid extrusion by phase-contrast microscopy. Samples were counted blindly, scoring more than 350 parasites per sample.

### Microneme Mic2, Mic8, and Mic10 secretion by Western blotting.

Microneme Mic2, Mic8, and Mic10 secretion by Western blot was performed as published (31). Freshly lysed parasites treated ± ATc for 48 h were resuspended in DMEM/FBS and added to a 96-well round-bottom plate, and secretion was induced by 1 μM A23187 or DMSO for 5 min at 37°C. For constitutive microneme secretion, there was no stimulation at 37°C for 60 min. Supernatants were probed by Western blotting using MAb 6D10 anti-Mic2 (51), rabbit anti-Mic8 (53), rabbit anti-Mic10 (57), and MAb anti-Gra1 (58). Signals were quantified using a densitometer.

### Microneme Mic3, Mic5, and Mic8 secretion by IFA.

Mic3 (30), Mic5 (52), or Mic8 (53) IFA on parasites exposed to a host cell monolayer was performed as published (31). Parasites resuspended in Endo buffer were spun onto HFF cells in a 6-well plate (28 × *g*, 5 min, room temperature) and incubated at 37°C for 20 min. Endo buffer was replaced with a mixture of DMEM, 3% FBS, and 10 mM HEPES (pH 7.2) and incubated at 37°C for 5 min. PBS-washed coverslips were fixed with 4% formaldehyde–0.02% glutaraldehyde followed by IFA in the presence of 0.02% saponin.

### Motility assessments.

Motility was analyzed by video microscopy essentially as described previously (14). Intracellular tachyzoites grown for 96 h ± ATc were physically harvested, resuspended in modified Ringer’s medium, and added to HFF confluent glass-bottom culture dishes (MatTek). The dish was imaged using a 63× objective at 37°C. Videos were recorded with 1-s intervals. Velocities of individual invasion events were analyzed using the ImageJ/FIJI Cell Counter plug-in.

### Moving junction formation.

Moving junction formation was determined as published (40) with previously described modifications (9). Parasites grown ± ATc for 48 h were inoculated into a HFF confluent 24-well plate by centrifugation (28 × *g*, 5 min, 20°C) and incubated at 37°C for 10 min. Wells were rinsed twice with PBS, fixed with 4% PFA at 4°C, and partly permeabilized with 0.02% saponin. MJ was detected using rabbit anti-RON4 (7), and all parasites were detected following full permeabilization with MAb anti-SAG1 DG52.

### Evacuole assay.

Evacuoles were determined as described (59) with modifications. A total of 1 × 10^7^ parasites grown ± ATc for 48 h were inoculated into HFF confluent 24-well plates. The plate was centrifuged (28 × *g*, 15 min, 23°C) and incubated at 37°C for 10 min. Wells were rinsed twice with PBS, fixed with 4% PFA at 4°C, and 0.25% Triton X-100 permeabilized. Evacuoles were detected by MAb Tg49 anti-ROP1 (54). More than 100 events per sample per experiment were counted.

### Immunoelectron microscopy.

Following washing with PBS, overnight-infected HFF cells were fixed in 4% PFA in 0.25 M HEPES (pH 7.4) for 1 h at room temperature and then in 8% PFA in the same buffer overnight at 4°C. They were infiltrated, frozen, and sectioned as previously described (60). Sections were immunolabeled with anti-Myc 9E10 in 1% fish skin gelatin and then with goat anti-IgG antibodies, followed by 10-nm protein A-gold particles before examination with a Philips CM120 electron microscope under 80 kV.

### Transmission electron microscopy.

Parasites were fixed in 4% glutaraldehyde in 0.1 M phosphate buffer (pH 7.4) and processed for routine electron microscopy (61). Briefly, cells were postfixed in osmium tetroxide and treated with uranyl acetate prior to dehydration in ethanol, treatment with propylene oxide, and embedding in Spurr’s epoxy resin. Thin sections were stained with uranyl acetate and lead citrate prior to examination with a JEOL 1200EX electron microscope.

### Statistics.

Student’s paired *t* test and one-way analysis of variance (ANOVA) using *post hoc* Bonferroni correction were used where indicated against the TaTiΔKu80 line.
