# Supplementary figures and images for: A Member of the Ferlin Calcium Sensor Family Is Essential for Toxoplasma gondii Rhoptry Secretion
Source: mBio. 2018 Oct 2;9(5):e01510-18. doi: 10.1128/mBio.01510-18 (PMC6168857; doi:10.1128/mBio.01510-18)

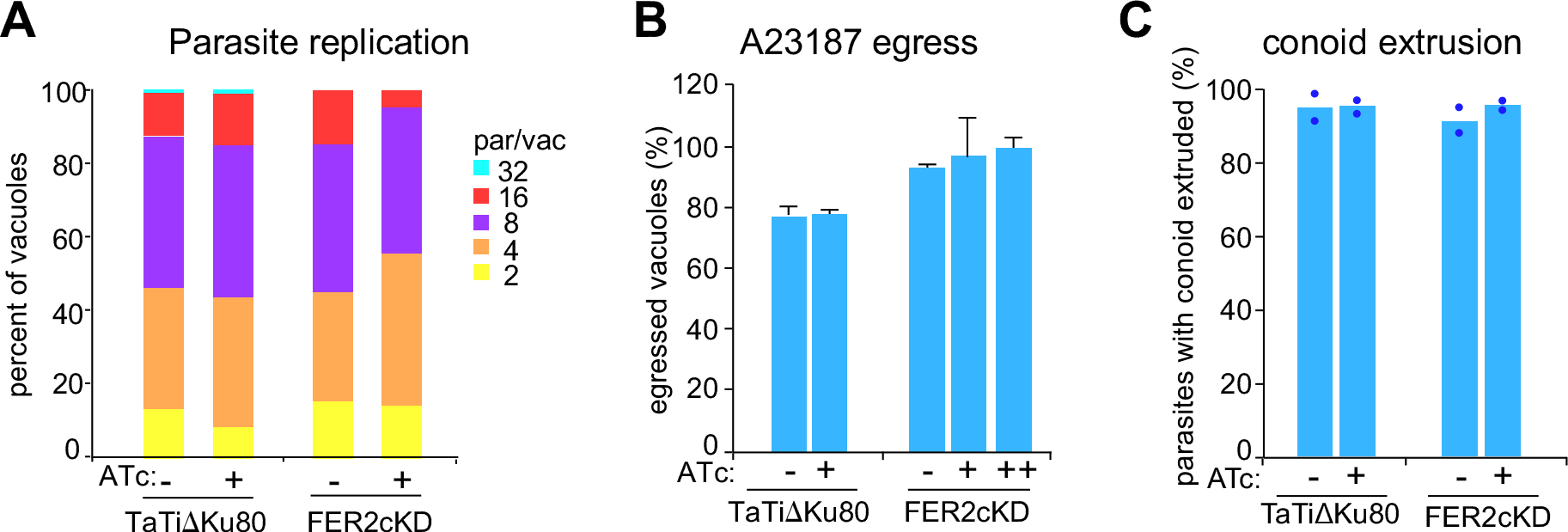

Supplement: FIG S1 [file mbo005184082sf1.tif]

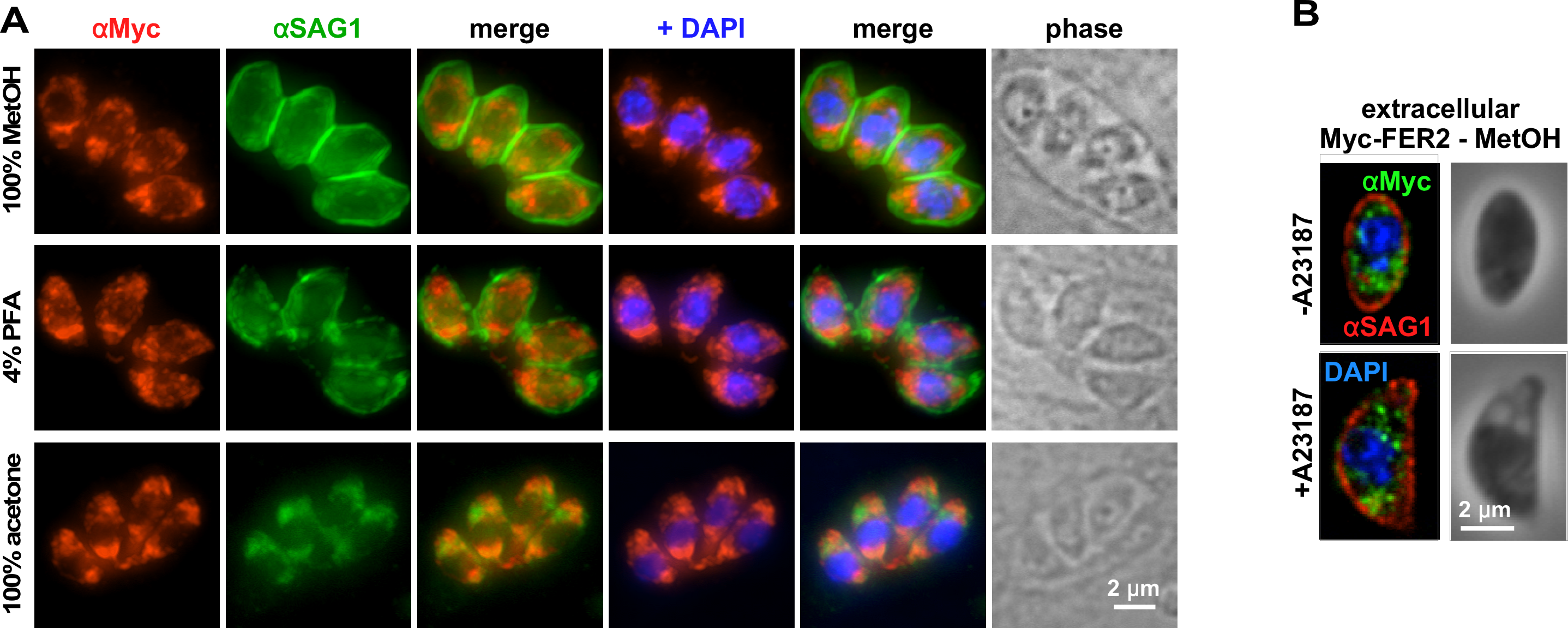

Supplement: FIG S2 [file mbo005184082sf2.tif]

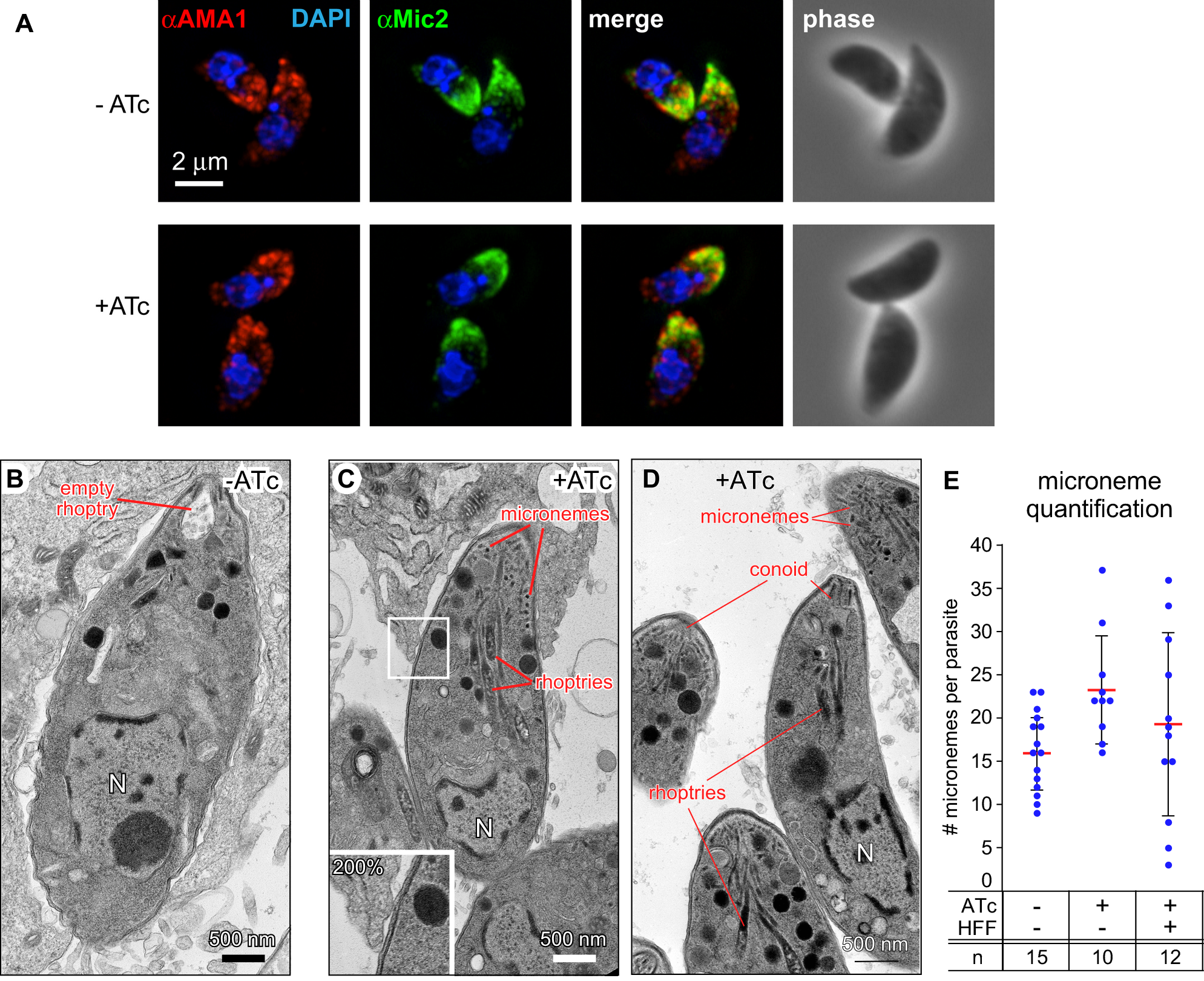

Supplement: FIG S3 [file mbo005184082sf3.tif]

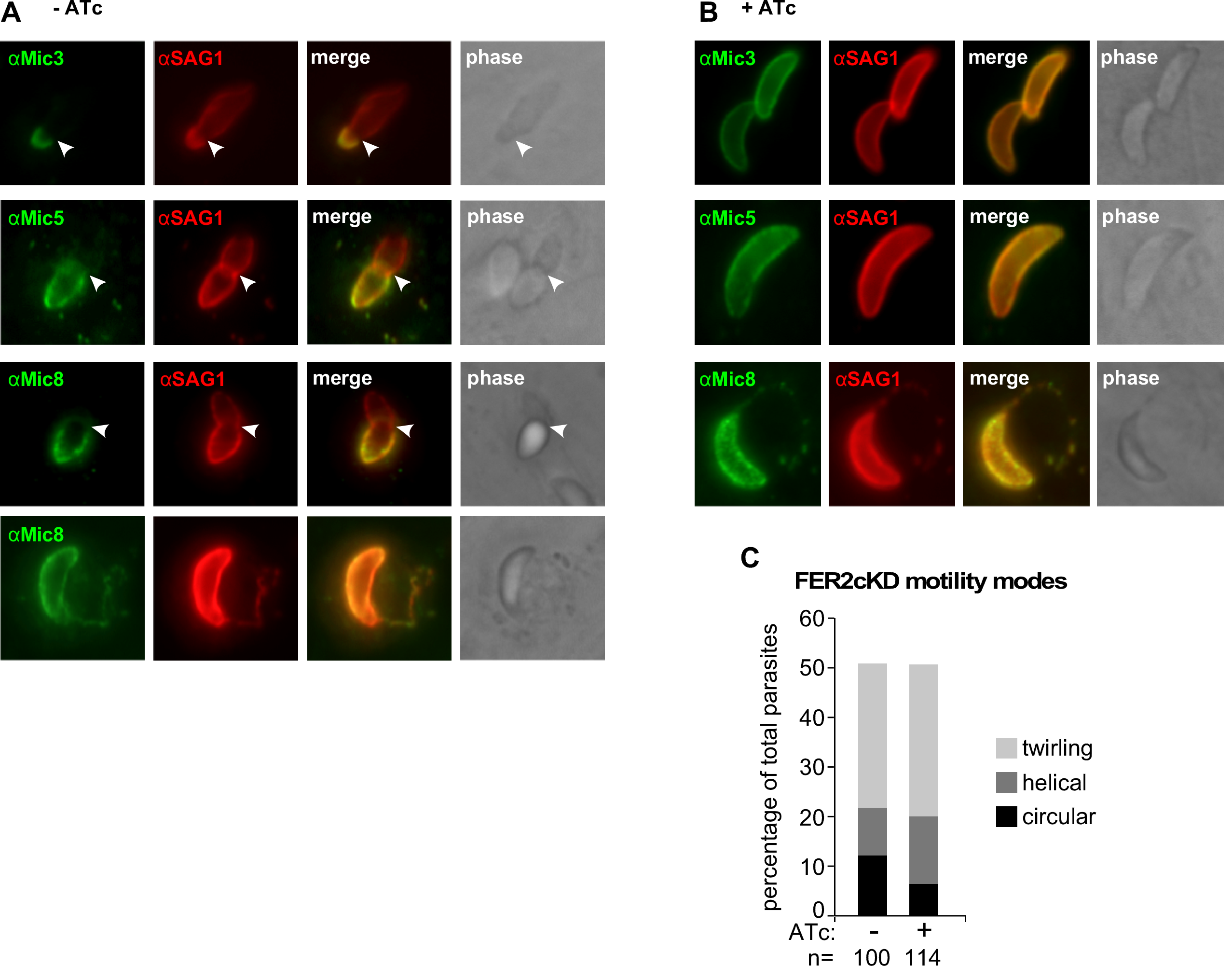

Supplement: FIG S4 [file mbo005184082sf4.tif]

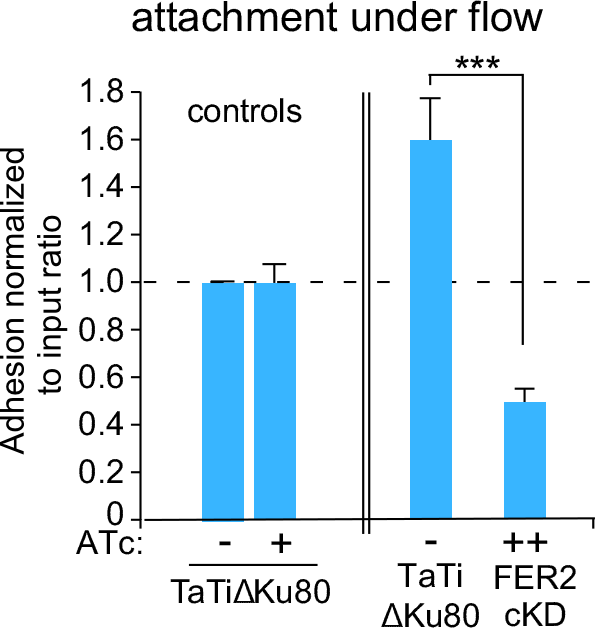

Supplement: FIG S5 [file mbo005184082sf5.tif]

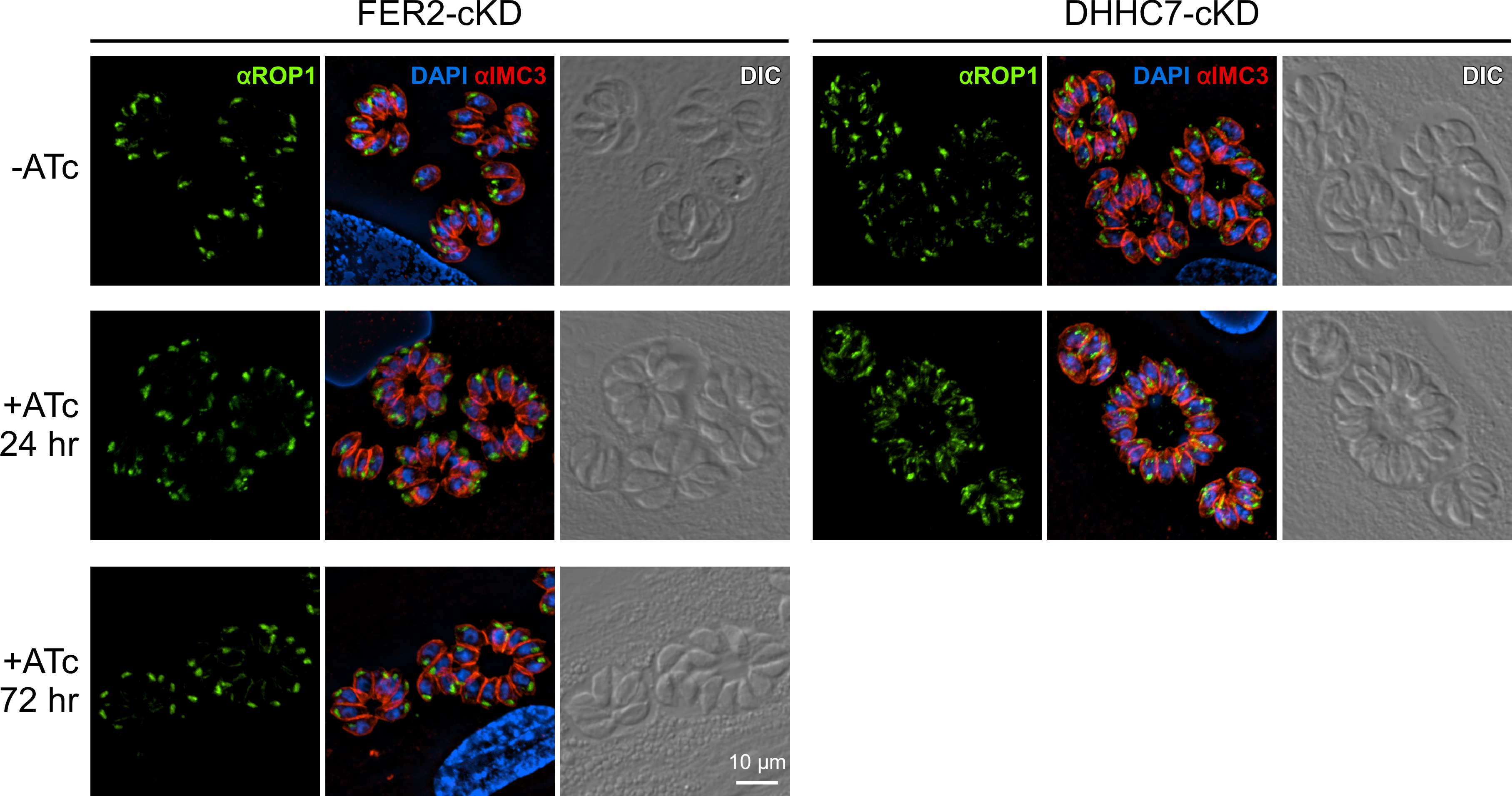

Supplement: FIG S6 [file mbo005184082sf6.tif]

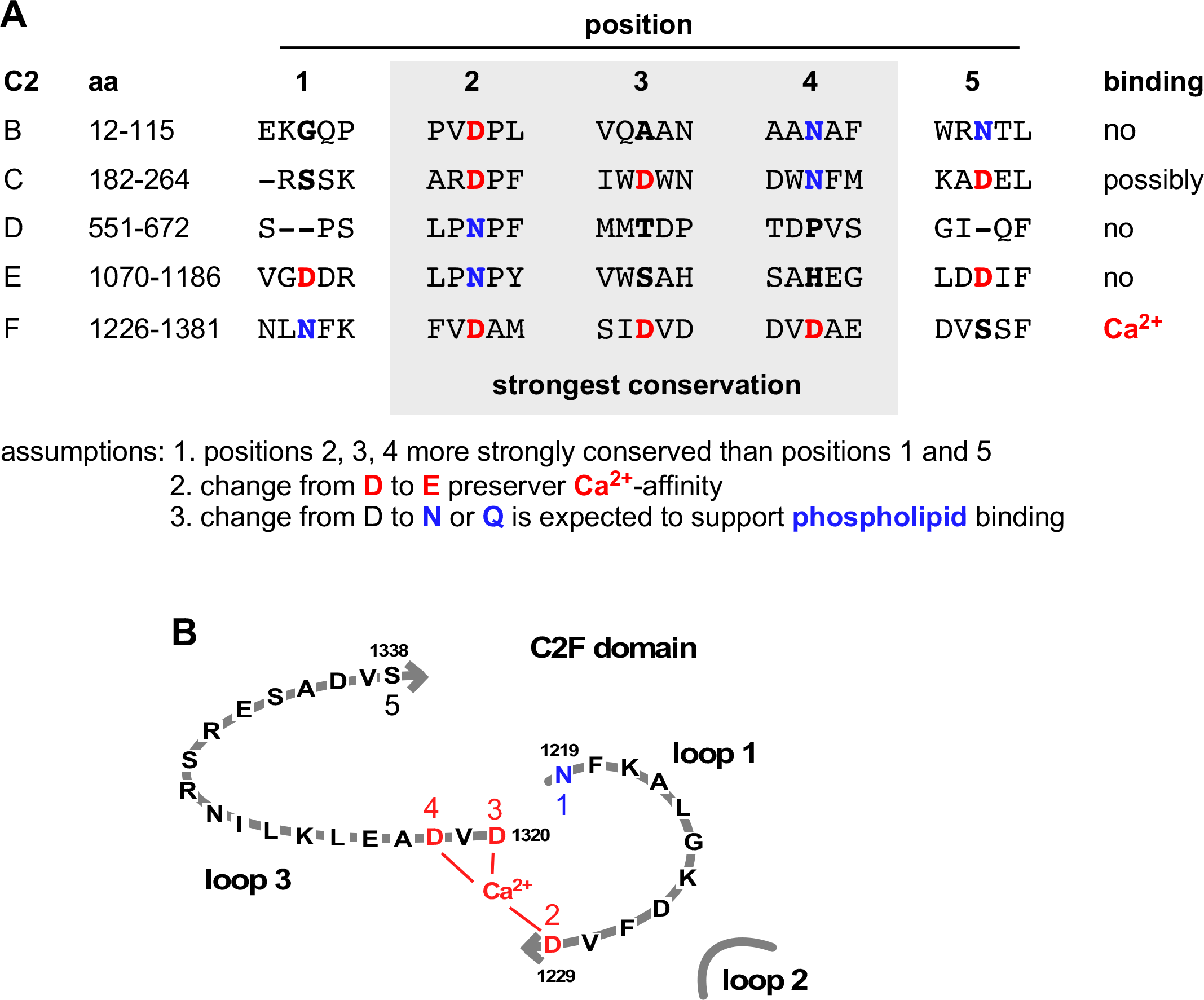

Supplement: FIG S7 [file mbo005184082sf7.tif]
