# Supplementary material for: A Member of the Ferlin Calcium Sensor Family Is Essential for Toxoplasma gondii Rhoptry Secretion
Source: mBio. 2018 Oct 2;9(5):e01510-18. doi: 10.1128/mBio.01510-18 (PMC6168857; doi:10.1128/mBio.01510-18)
Supplement: TABLE S1 [file mbo005184082st1.docx]

**TABLE S1** Primers used in this study. Restriction enzyme sites are underscored.

**Name Sequence**

*Sequencing primers to validate splicing and gene model*

YFP-F-seq aaccactacctgagctaccagtcc

FER2-seqF2 GTCGTGACACCGCTTCCAG

FER2-seqF3 GTCGTGACACCGCTTCCAG

FER2-seqF4 CTGTCTCTTCATATGCCTCGG

FER2-seqF5 CGACGATGTCATTTTCCCTT

YFP-FER2_R_RV ggtccGATATCCTAACCCAGCAGCGAGGGATAGAG

*FER2 expression and tagging constructs*

YFP-FER2_F_NheI ggtccGCTAGCATGGGTAAAACTAAAATTTACTCGGTTGGTTTCACC

YFP-FER2_R_EcoRV ggtccGATATCCTAACCCAGCAGCGAGGGATAGAG

FER2-LIC-F TACTTCCAATCCAATTTAATGCTGACGCTAACCTTGACGCTTGTAACG

FER2-LIC-R TCCTCCACTTCCAATTTTAGCACCCAGCAGCGAGGGATAGAG

*Generation and verification of FER2-cKD parasites*

BamHI-FER2_F GGTCCggatccATGGGTAAAACTAAAATTTACTCGGTTGGTTTCAC

NotI-FER2_R GGACCgcggccgcAATCGCGGGAAGACACGAACG

T7S4FER2-int-F CAGGTTTAAACCACTAGTTCTAGAAGGACCCGGTAC

FER2-int_R CTGCTTTGCGAAGATCATCGTAGTCG

pFER2-int-F GGGGACAAGTTTGTACAAAAAAGCAGGCTGCCTTTAATTCACACAT-GCGACAAGGGTCG

*Generation of DOC2-5xTY5*

5xTy_upstream_F TTCTGGCAGGCTACAGTGACACC

5xTy_PlusLink_R GGTCCGATATCTTACTTAAGGCCGTCAAGTGGATCCTGGTTAG

DOC2_3-target_F GGACCgtttaaaccctaggGAGCGAGACACTGCCCGAGAGC

DOC2_3-target_R GGTCCggatcccatatgGGTGCGCCCAGCCAGATCC

*Generation and validation of ts-DOC2 parasites (mutations in lower case)*

DOC2_proto_F AAGTTGCGACAACTTCTCCGCCTTCAG

DOC2_proto_R AAAACTGAAGGCGGAGAAGTTGTCGCA

DOC2_FM>SV_F GGAACCTGCAAATTCAAAACTACATGGACGACAACTTCTCCGCCT-cCgttGAGTTCGAGTTCGGAGGCACACGCGAAGAATGCAAAGTCC

DOC2_FM>SV_R GGACTTTGCATTCTTCGCGTGTGCCTCCGAACTCGAACTCaacGgAG-GCGGAGAAGTTGTCGTCCATGTAGTTTTGAATTTGCAGGTTCC

DOC2-5'UTR_F GCTCTACTCGTGTACTACTCTGAGCC

DOC2-intron3_R GCGGACCTCCTGATACCATGC

DOC2_F>S_seq Atgcatctgttggtggagg
